# A gender perspective on perceived causes of sick leave due to common mental disorders among young Swedish employees: A qualitative interview study

**DOI:** 10.1371/journal.pone.0291551

**Published:** 2023-09-18

**Authors:** Caroline Olsson, Helena Tinnerholm Ljungberg, Elisabeth Björk Brämberg, Lotta Nybergh, Irene Jensen

**Affiliations:** Institute of Environmental Medicine, Karolinska Institutet, Unit of Intervention and Implementation Research for Worker Health, Stockholm, Sweden; Public Library of Science, UNITED STATES

## Abstract

**Objective:**

Common mental disorder (CMD) is the most common reason for sick leave among young employees in Sweden, with young women having a higher prevalence. There is a lack of studies focusing on young employees’ own perceptions of sick leave. The aim was twofold: to investigate 1) perceived causes of sick leave due to CMD among young employees, and 2) differences and similarities among women and men.

**Methods:**

Using a qualitative design with an applied gender perspective enabled us to capture young employees’ gendered experiences and consider cultural and social aspects of their situations. We interviewed 13 women and 12 men (aged 20–29) with experience of being on sick leave and applied a conventional inductive content analysis.

**Results:**

Six categories were identified: a) Being new to the labour market and the workplace; b) Want to prove themselves; c) To be exposed to poor working conditions; d) Relations at work; e) Being vulnerable; and f) Additional private life burdens.

**Conclusion:**

This study adds to the understanding of young employees’ perceived causes of sick leave due to CMD, by letting them share their experiences of events prior to sick leave connected to work and private life. Similarities and differences in women’s and men’s experiences were revealed. Overall, both young men and women describe a more pressured work situation for young women compared to their male counterparts, that young women take more social responsibility in private life and at work whereas men on the other hand find it harder to disclose mental health problems.

## Introduction

Common mental disorders (CMD), i.e. depression, anxiety, adjustment disorders and stress-related ill health [1] have been a long-lasting problem in high income countries, affecting the individual, workplaces, healthcare and society at large [2,3]. In terms of sex, it can be concluded that young women have a higher prevalence of CMD than young men [4,5] and that this association is cross cultural and has persistent over a long period of time [6]. To enter the labour market is a vital part of the transformation from adolescence to adulthood [7], and getting a good start in work life is essential for future work life and mental health [8]. However, poor work environment including work with low control and high work load [9,10] as well as poor conditions on the labour market, such as work during atypical working hours [10], precarious employment [11,12] and the risk of unemployment [13–15] are frequent for young workers and are also known risk factors associated with mental health problems [11–13,15]. An early onset of CMD is, furthermore, associated with mental health problems later in life [16] and initial problems at the labour market can affect possibilities to make a career further on in life [17]. In contrast to other diagnoses, sick leave due to CMD is more common among young adults in many Western countries [5,18,19], including Sweden [13], and is the most common sick leave diagnoses among young workers in Sweden [13,20,21].

An applied gender perspective [22] combined with an awareness of how gender norms can intersect with young age can be beneficial to understand why young women have a higher risk of being entitled sick leave benefits due to CMD than young men [23]. Since gender can be understood as a ‘fundamental organizational principle’ of societies that varies over time and contexts [24], perceptions of gender among young adults today may differ from other age groups or previous generations. These perceptions can be understood in terms of gender norms and expectations, stating how men and women “ought to be” [25]. Gender norms uphold the gender order [26], also known as gender system. Gender systems are not static, however most gender systems have been unequal where men and masculinity has been privileged and higher valued compared to women and femininity [25]. Even if gender mainstreaming and policies against gender discrimination have been developed in Sweden and in the EU, discrimination in workplaces still occur. Therefore, working life is one important context for (re)creation of gender norms and is a part of the gender order [27]. Gender norms also contribute to an unequal distribution of men and women in different work sectors [28] where gender norms are restricting both women’s and men’s career choices [29] and generate different working conditions for young men and women.

An often-stated cause for women’s higher risk for sick leave is the horizontal gender segregation, i.e. that women and men are over- or underrepresented in different work sectors, occupations or workplaces [30]. The female-dominated occupations in the education, health and welfare sectors are characterized by well-known risk factors for sick leave, such as high demands, low control and an imbalance between effort and reward. The vertical gender segregation also contributes, i.e. that men more often have leading positions with greater opportunities for control over their own work situation [31–34]. In Sweden, a dual earning model has been promoted for decades and women’s participation in the labour market is one of the highest in the world [35,36] made possible by a large supply of public childcare facilities. Even if the proportion of females in the working population is high, it is still more common that women work part time to reduce the hours that children need to be in day care [36]. The Swedish labour market is also strongly horizontally segregated between the sexes, where women work in the public sector with health care or in teaching to a more often than men [27,28].

Qualitative studies [37,38] have explored the complex processes of how contextual, organisational, and individual factors, in intertwined ways, contribute to workers’ need to be on sick leave due to mental disorders. Holmlund et al [37] applied a gender perspective to demonstrate that everyday life events situated at work and in private life taken together add up to reasons for sick leave due to CMD, and that there are gendered processes involved in these events. Both sexes, in the ages of 24–55 years, identified high workload and poor work conditions as central reasons for their sick leave. However, women took more emotional responsibility for creating and managing relations and for caregiving, both at work and in private life, which was perceived as adding to their mental strain.

The reasons behind the high numbers of sick leave among young workers needs to be further examined, especially since early onset of CMD tends to be followed by mental health problems later in life [8]. Furthermore, we need additional studies that explore differences between young women and men, given the sex discrepancies among young adults in sick-leave rates due to CMDs. Hence, we believe that a study focusing on the intersection between young age and gender, could help us to explore perceived causes sick of leave due to CMD among young men and women. Using a qualitative approach with an applied gender perspective enables us to describe these perceived causes and take into account cultural and societal aspects of the participants’ situations, with a focus on young age and gender [39]. The aim of this study was twofold: to investigate 1) perceived causes of sick leave due to CMD among young employees, and 2) differences and similarities among young women and men.

## Methods

### Study design

This manuscript reports findings from an ongoing project, for which full project details are described elsewhere [40]. A qualitative research approach was chosen to aim for rich and nuanced descriptions of the young employees’ experiences of work, CMD and causes of sick leave [39,41]. In this study, a gender perspective, focusing on the intersection between young age and gender, was applied to all parts of the study, including the aim, sampling, and analysis.

### Setting and procedure

**Fig 1:**
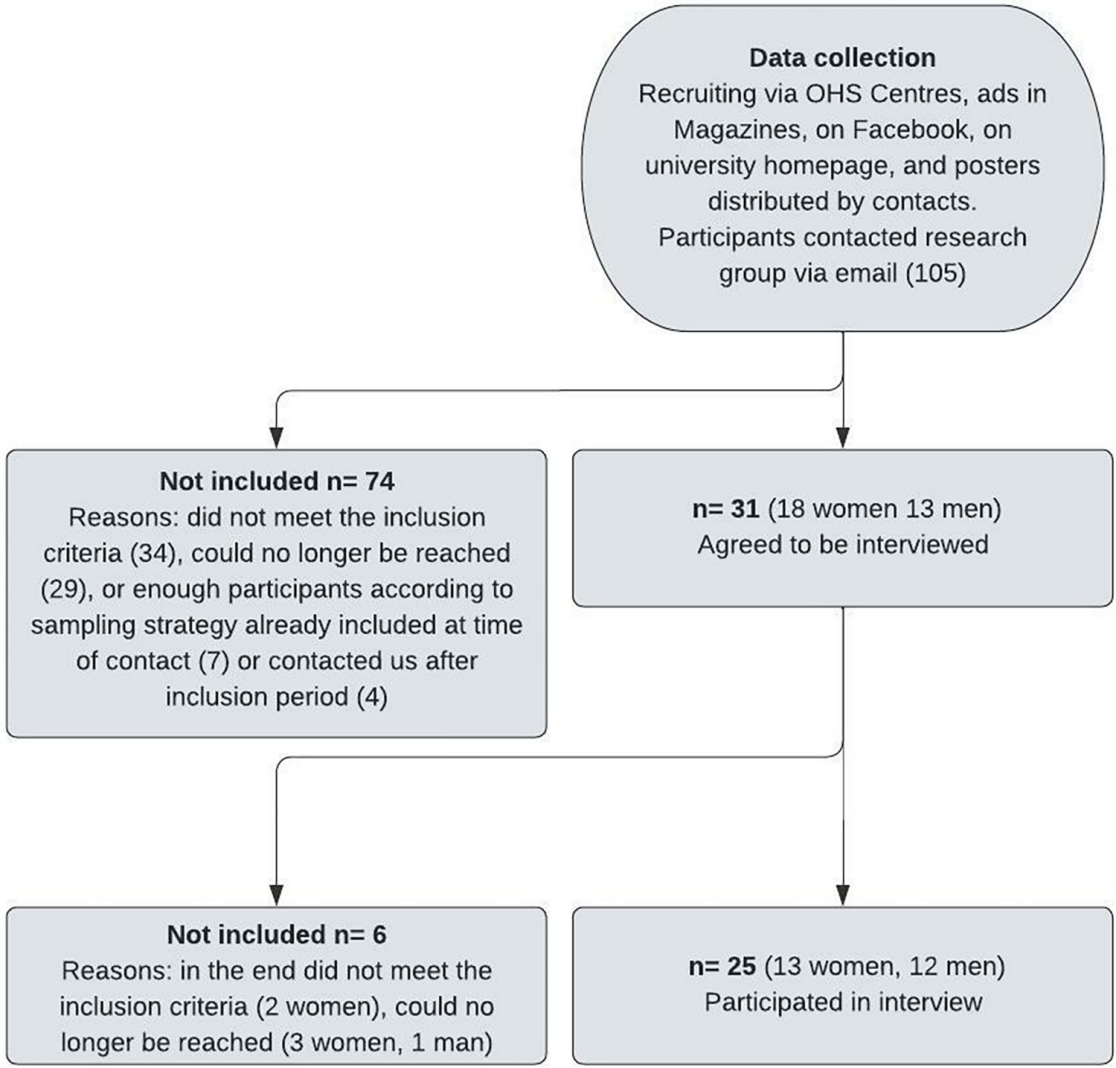
Flowchart of the recruitment of participants.

This study was conducted in Sweden and participants were recruited nationally using a purposeful sampling. National recruitment was made possible through the use of video platforms (Zoom and MS Teams) for data collection [42]. A few participants chose to do the interview by telephone. The participants were recruited from October 2020 to May 2021.The young adults were reached via ads on university homepages, in magazines and on Facebook Furthermore, posters and flyers were distributed to contacts of the researchers, and by OHS centres to broaden the outreach. Ads, posters and flyers were designed in a gender-neutral way, however, nobody that identified themselves as non-binary or having a gender other than male or female contacted the research team. The recruitment material contained information about the research project, the inclusion and exclusion criteria and how it was possible to participate. Those who were interested in participating, sent an e-mail to the research group. Then CO and HTL called the person to verify the inclusion criteria and book an interview. For a flowchart of the participants, see Fig 1.

The inclusion criteria for the young employees were that they 1) at the time of the interview were 19–29 years old, and 2) currently were, or had been within the past two years, on sick leave for 3–12 weeks due to CMD. The exclusion criteria were 1) co-morbidities that could severely affect the experiences of the sick leave; 2) individuals suffering from post-traumatic stress syndrome, neuropsychiatric diagnoses, psychotic symptoms, or bullying problems; and 3) persons without employment or employed by employment agencies at the time of their sick leave.

### Data collection

The data collection resulted in 25 individual semi-structured interviews [43,44]. The participants completed a web-based questionnaire before the interview that included questions about their self-perceived gender, age, household composition, highest completed education, time employed at their current workplace, and mental health measured with the Hospital Anxiety and Depression 14-item scale, as well as with the s-UMS [45,46]. Their age range was 20–29 with the median age being 27 years old. None of the participants identified themselves as non-binary or a gender other than male or female; 13 women and 12 men participated. These and other participant characteristics are presented in Table 1.

**Table 1:** Personal characteristics.

	Women (n 13)	Men (n 12)
**Age (mean, range)**	26.77, 20–29	26.75, 22–29
**Level of education (n)**		
Primary/secondary education	4	8
Higher education/university	9	4
**Years at the current workplace**		
<1	2	6
1–2	5	2
3–5	6	3
6–10	0	1
**Work sector**		
Health care/Social services/Education	6	3
Service	2	3
White collar	5	1
Blue collar	0	5
**Living conditions (n)**		
Alone	7	8
Living with parents	1	0
Living with partner	4	3
Living with partner and child	1	1
**Hospital Anxiety and Depression Scales (HADS)**		
Anxiety		
No Anxiety	1	1
Mild Anxiety	8	4
Anxiety	4	7
Depression		
No depression	9	4
Mild depression	2	4
Depression	2	4
**Self-rated Exhaustion Disorder (s-ED)**		
No s-ED	6	5
Mild s-Ed	4	3
s-ED	3	4

### Interviews

The research group designed a semi-structured interview guide, in Swedish, based on the aim of the study. Two pilot interviews, one of each sex, was conducted by CO and HTL to test the guide. The pilot interviews led to a few minor changes. The interview guide contained questions regarding perceived causes of sick leave, probing questions about work, home conditions and lifestyle. In addition, there were specific questions about gender and age in relation to sick leave due to CMD:

- Based upon what we have talked about so far, would you say that it is important that you identify as woman / man / other gender category as specified by respondent in web survey (follow-up questions related to the project’s gender perspective on these issues)?
- Based upon what you said today, would you say your age matters in any way? And if, how?

The entire interview guide is published as supplementary material for our study protocol [40]. All interviews were conducted by CO or HTL, both of whom are trained and experienced in qualitative interviewing. CO and HTL had daily meetings during data collection to make sure that the interviews were conducted in a similar way. The duration of the interviews varied between 34 minutes and 86 minutes. All together it was almost 24 hours, and the mean time was 58 minutes. The collected data will also be used in two other articles with focus on return to work and on the effects of Covid 19 to remain at work. The audio recordings were transcribed verbatim by a professional company and then pseudonymised.

### Research team and reflexivity

To design and conduct a research project is always related to choices made by researchers. This applies to the aim and research questions, methods and theories and how well these choices are implemented in the project. We chose to conduct a qualitative interview study to let the young adults use their own words when describing their experiences of CMD and sick leave. Furthermore, we chose to apply a gender perspective to better understand the living conditions for young men and women in Sweden, a country with high ambitions of gender equality but with a gender segregated work force. We understand that our preknowledge and research background have framed our understanding and way to investigate the young adults’ experiences. Challenges in the project were discussed in the research group to combat any biases caused by issues regarding to power imbalance. The research group consists of individuals from different academic disciplines, including health sciences, sociology, and gender studies. We believe that these open discussions with different perspectives strengthened the reflexivity and improved the quality of the study [47,48]. Interviews are a commonly used method in qualitative research to gather data with the purpose of capturing perceptions and experiences from participants. It has many advantages, but some challenges should be noted [41]. Research interviews are affected by power relations, where the researcher often has more status and a higher educational level than the participants [49]. Moreover, it is a delicate matter to ask sensitive questions of young adults with experience of CMD, however the participants had volunteered, and they shared their stories using their own words which made it possible for them decided what they wanted to share and not.

### Ethics

This study was approved by the Swedish Ethical Review Authority (registration number 2020–03271). Written and oral information about the project was given to those eligible for participation. They were informed that their participation was voluntary and that it was possible to withdraw from the study at any time without stating any reason. Informed written consent was collected from all participants. The project was funded by the Swedish Research Council for Health, Working Life and Welfare (Forte; grant number 2019–00883). The founding body did not have a role in the study.

### Data analysis

The analysis process is described in detail in the study protocol [40]. Conventional inductive content analysis was applied, meaning that codes were derived from the data and not determined beforehand. We first coded all data regardless of gender and then compared statements for men and women [50]. To facilitate the process, Nvivo software [51] was used. The research group read several of the interviews and CO and HTL read them all numerous times to get a broad sense of the data. Then CO and HTL coded the first five interviews together to get a common view of the material and the coding structure. The rest of the interviews were coded by either CO or HTL independently. Throughout the process, the research group jointly discussed the coding to improve credibility. Short summaries of each participant’s interview were written directly after the coding, to contribute to the understanding of how codes were linked and to better understand the overall process of sick leave due to CMD, as described by the participants. Short notes comparing the statements of female and male participants were made. During the analysis process, codes that were similar were merged into sub-categories, and the sub-categories were further abstracted into main categories [52]. Thereafter, all categories were discussed within the research group and statements from men and women were further compared to deepen the gender analysis. In this part of the analysis, similarities and differences between the sexes and work sectors were noted. Moreover, all statements of the participants and the categories we created were discussed in relation to gender norms.

## Results

The analysis revealed six main categories and 16 sub-categories in total, presented in Table 2 and identified gender differences are marked with an asterisk. There were no distinct patterns identified due to work sectors. Quotations from the interviews provide a link to the data and serve as illuminating examples of participants statements.

**Table 2:** Overview of the results; Main categories and sub-categories.

**Being new to the labour market and the workplace**
Given too much responsibility and too difficult tasks when new at work
Fear of not fitting in socially*
**Want to prove themselves**
High level of ambition and expectations on oneself*
Expectations on young employees to be healthy and capable*
**To be exposed to poor working conditions**
Boundaryless work*
Care and nursing work with responsibility for others is demanding
The employer has poor working conditions
**Relations at work**
Lack of support from colleagues and manager
Conflicts at work*
Gender-based vulnerability*
**Being vulnerable**
Previous experiences of CMD
Did not understand how ill I was*
**Additional private life burdens**
Activities besides work add to the overall pressure
Responsibility for care of relatives is a burden*
Problematic upbringing and personal tragedy
Poor romantic relationships*

*Differences between young women and men.

### Being new to the labour market and the workplace

Given the participants young age, many were new to the labour market and at their current workplace. They talked about this situation as challenging and described how their limited life and work experience left them unprepared about how to act in this new context, both socially and in terms of workload and content.

#### Given too much responsibility and too difficult tasks when new at work

The participants recounted that they thought that the employer should have taken more responsibility, with better introduction and guidance. These experiences were described in a similar way by male and the female participants. The need of better introduction was pointed out by this participant:

I do not feel that I received a good introduction compared to other jobs. The introduction given at other places is more structured and you are given clear instructions and you get like… well, maybe papers to read through, while here it was more like "Yes, open the system and play around a little”. (IP 8, woman, debt collector agent, white-collar worker)

Instead of support, when being new at the workplace, they often felt alone without guidance from senior colleagues or managers. Central in the descriptions was also that, too early in this new situation, they felt that they had been given difficult work tasks with too much responsibility for their juniority. These high demands were difficult to meet and led to feelings of inadequacy and stress. These perceived demands were especially brought up by female participants and was strongly emphasised. There were descriptions on how there was no room for mistakes or errors, as illustrated by the following quote:

But somehow, from day one, a lot of responsibility was placed on me. And a lot of expectations. And it’s not just… It’s not that you are expected to work a lot, but they expect you to be extremely effective every minute of the day while at work. You’re not allowed to have a bad day. (IP 19, woman, accountant, white-collar worker)

#### Fear of not fitting in socially *

In addition, some of the women, but none of the men, also expressed worries about not fitting in socially at the workplace, particularly at the beginning of employment. This was not expressed as a sole cause, but in addition to high work demands it was perceived to contribute to the overall burden:

But it was probably due to it being a new job as well as you are not really sure where you fit in and "Who am I in this group?", yes. So it was as if you needed to find your role (IP 2, woman, employment consultant, social services)

The social part of work was also related to creating good relationships and taking care of colleagues, as expressed in the following quote:

If you imagine me at my workplace. I’m very good at making sure that I’ve talked to everyone, I can talk to everyone, I make sure that everyone feels good, that sort of thing. I think that, if I compare it with my older colleague and my close colleague who is a man, they don’t have the same kind of… well, behavior. It’s also the case that my older colleague who is a woman, she says "Yes, but you keep track on everything and so on. I don’t understand how you manage." And that’s also something I’ve cut back on. You don’t have to keep track of everything and everyone and make sure that everyone feels good, here and there. (IP 9, woman, occupational therapist, health care)

### Want to prove themselves

The young employees shared stories about how they wanted to prove themselves as competent and capable. Moreover, they did not want to be a nuisance in the workplace and need support and assistance.

#### High level of ambition and expectations on oneself *

The participants talked, to a large extent, about how they viewed themselves as highly ambitious, using phrases such as ‘having (too) high expectations of myself’, ‘to prove oneself’ and ‘be driven’. To be ambitious was often described as something that the young person ‘had always been’, and it was attributed to different social contexts, such as school, in private relations, and at work. Still, it was in combination with the current employment that this drive became a burden and was described as a cause of sick leave. There were male participants who expressed these thoughts, but to a greater extent, they were emphasized by the women. For some it was a described as a struggle with gender norms:

It is this typical view of women. That they are more fragile, cannot cope with as much and yes. And I think that has also contributed to the pressure I feel because I want to perform and show, that yes, I am not the stereotypical woman. I can do more. That in itself may have put more pressure on me, right there and then two years ago… (IP 12, woman, after-school leisure teacher/assistant nurse, education/health care)

At the same time, the participants described how they had trouble identifying what could count as ‘good enough’ at work. In addition, participants lacked the experience to set limits and prioritize their duties, and stated a lack of guidance from managers in setting these limits:

You want to prove yourself and you want to perform, and you are not very experienced. You have not understood that you have to draw boundaries and where to draw them. (IP 21, man, business analyst, managerial position, white collar-worker)

This combination of having high ambitions and no experience of setting limits was identified by the participants as a cause of sick leave, since it made it difficult for them to understand when things got out of hand and when this drive became a contributing cause of their mental health problems. The drive and high ambitions were more frequently described as being something ‘female’, and both men and women described this attributed gender difference as a trait more common among women.

#### Expectations on young employees to be healthy and capable *

Moreover, the participants’ self-image of being young, included characteristics of being ‘healthy’, ‘strong’ and even ‘immortal’, which applied for both women and men:

And maybe I thought that I was young and immortal as well. That there would be no risk in me going full-on. (IP 16, woman, real estate manager, white-collar worker)

The above described self-image of being young, strong and healthy was reinforced by social norms at the workplace and it was attributed to both men and women. The participants described how they felt questioned when they told others about their mental health problems. Both female and male participants felt that it was even harder for men than for women to talk about their mental health and this was contributing to their risk of CMD:

But being on sick leave for mental health reasons at the workplace, for me anyway, felt more stigmatized. And for me as a young adult, I feel that I want to show that I am capable and can take responsibility and manage on my own. (IP 25, man, teacher assistant, education)

### To be exposed to poor working conditions

Being young meant a lack of experience in setting boundaries or prioritizing. Young people did not know how much it was reasonable to work and felt that their employers did not set these limits either.

#### Boundaryless work *

The young workers described a demanding work situation with stressful schedules and various aspects of boundaryless work. It was common with long working hours, high workload, work out of office hours, expectations on checking e-mails or having their phones switched on after office hours. This was reported to create stressful feelings just to know that you might have unattended e-mails or that you were not able to finish your duties during worktime. There were many descriptions of when the participants working long hours:

Because we work very, very much from January 1st until the end of June. On a quiet week we might work 55 hours a week. And at most I worked up to 90 hours, my last week. But on average I was working around 70 and 80 for quite a lot of weeks. (IP 19, woman, accountant, white-collar worker)

In addition, there were little or no time to recover during working hours. Participants from different types of jobs, like health care, retail and restaurants, described how they did not take breaks or have time to eat, even when they worked long hours:

It was very stressful, and I could work 16 hours a day and there were no breaks and if you wanted to eat you had to stand up and eat while you were working. (IP 15, man, chef, service)

Both men and women talked about too much workload but experiences of boundaryless work were expressed more often by female participants than the males and were connected to their high levels of ambition. That made it difficult for them to let go of work off-hours and made them worry about work in a boundaryless way as expressed in the following quote:

I have a hard time letting go of the job when I get home, just this desire to perform, especially when you are new. I didn’t really know what was expected of me, and that makes you take on more than you can possibly carry. (IP 2, woman, employment consultant, social service)

#### Care and nursing work with responsibility for others is demanding

Another experience was to have too demanding care responsibilities at work, which was expressed by those employed in schools, care services and the healthcare service regardless of gender. The young employees in these sectors thought that they lacked time and sometimes knowledge on how to deal with pupils or patients’ needs. There were also descriptions of how pupils needed more support than the young employee could provide, and which went beyond the job description. This led to feeling of insufficiency in relation to other human beings’ welfare and that was emotionally exhausting according to the participants.

The actual job description is that we are after-school leisure leaders, but when we work with young people and children, there is much more you do. You’re really not just a youth worker, you’re sometimes a sibling, a parent. You’re really everything in between. And recently, or even then, children and young people who were having a hard time at home and who had some… What is it called? They had… It was mental illness that many of the children had. Because of various things. Some were because of school and others because of family. And these were things that we needed to address. Because they had confidence in us. And sometimes you didn’t always have these external parties to pass them on to. (IP 12, woman, after-school leisure teacher/assistant nurse, education/health care)

#### The employer has poor working conditions

To be constantly exposed to high workload due to lack of resources was part of the young adults’ poor working conditions, as perceived by the participants. Some also implied that high workload was part of the employers’ strategy, and that employers were known to take advantage of having young, unexperienced staff:

I think about the fact that they know that there are many unemployed young people who would consider working with this. So it does not feel like we are important as staff because they know that we are replaceable. (IP 23, man, caregiver, social service)

Adding to this situation was the fear of losing one’s job and this was seen in the light of young age where the participants talked about having to keep one’s job, getting merits on the CV. The fear of unemployment was further stressed by those with temporary employment. Men and women described these feelings of job-insecurity in a similar way, and they thought it was associated with their young age. To ask for help or to show yourself vulnerable was described as a risk not worth taking:

…then I had probationary employment for 6 months. I felt bad before the 6 months were over, but I tormented myself because I was so afraid that "if I go on sick leave now then I may not be allowed to continue", and that’s awful (IP 2, woman, employment consultant, social service)

### Relations at work

Being part of the workplace also involves social relationships where support from colleagues and managers is significant. Young employees talked about situations where they felt they lacked this kind of support.

#### Lack of support from colleagues and manager

The participants’ expectations of their managers leadership were not always met, when looking in the back mirror they could see that their manager had not set limits on the amount of work or hours worked. They further described that they would have needed more guidance in how to prioritize. Moreover, the participants thought they lacked support not only from managers but also from colleagues who were tied up with their own duties. Female participants especially described how they felt left alone, without supervision on how to conduct and plan their work tasks:

But what I would like to say is that I expected my employer to take more responsibility. And I did not know that I would be needed to set boundaries and speak out. I thought you would get a reasonable workload in a workplace. Especially in my entry-level job, somehow. (IP 19, woman, accountant, white-collar worker)

Another aspect in this category was that participants, when they started to feel mentally distressed, did not ask for help. They did not want to be seen as week or to take up the manager’s time, they just kept on working until they could not cope anymore. On the other hand, those who did ask for help felt that their manager either didn’t listen or provided insufficient support.

#### Conflicts at work *

There were also reports of problematic leadership in terms of bad behaviour from the manager. This was reported especially by male participants, who experienced aggression from their managers and gave examples of how managers yelled at them. Some men felt punished by their manager in terms of being assigned more work tasks than their fellow colleagues, without any reward for the extra efforts.

Especially those who know a little more…///… then we jump around between different parts of the company when… where it’s needed most, but then you never get any time to recover when it’s quiet. For those who only know one thing in the company, well, if there’s a lot to do for a while, then you can sit there, but then you have to stay there because you don’t know anything else. And then it becomes an enormous stress for us who are just moved around all the time, and "you can do that, then you have to move there when it’s most intense there and then there when it’s most intense there". And they don’t show any appreciation for that either, you… you don’t get any extra rest days. (IP 17, man, factory worker, blue-collar worker)

Lack of appreciation or positive feedback from the manager also made it harder to manage high workload:

It’s quite a stressful job, but it was not a problem for me, the stress at work. That was not the problem at all but how they treated their employees, that was the problem. There was no appreciation for what you did or how you did it. (IP 13, man, taxi-driver, blue-collar worker)

#### Gender-based vulnerability *

Some of the women, but none of the men, had experienced gender-related harassment at the workplace and also felt that managers did not deal with the problems when reported:

One of my colleagues was inappropriately groping me. So I talked to her [the manager]. And… What had they done? He [the colleague] was moved to another section of the after-school club, and that was it. And so he continued to work with young people, and it just happened… It was… It just escalated… And I thought that they could have done something more than just move him to another section and let him continue inappropriately touching young people. (IP 12, woman, after-school leisure teacher/assistant nurse, education/health care)

### Being vulnerable

Feeling extra sensitive in the past or experiencing mental health problems for the first time was described by young employees. Having had previous experience of mental health problems could be seen as both an enabling and hindering circumstance.

#### Previous experiences of CMD

Nineteen out of 25 of the young workers had previous experiences of CMD but most of them had managed to work or study anyway:

Yes, I think I have suffered from mental health issues since I was a child […] I have had depression before, I have, but it is not something I have gone on sick leave for. (IP 22, man, building caretaker, blue-collar worker)

The tipping point came in relation to their current job situation that was experienced as highly strained; however, these participants also described themselves as vulnerable and that they partly saw this vulnerability as a matter of personality. In some cases, previous experience of mental health problems made it possible to identify, at an earlier stage, that they were falling ill again:

I realized that "Hold on, now I recognize what’s going on." So then I felt that I could stop… It was important for me to stop it before it got worse. (IP 25, man, teacher assistant, education)

#### Did not understand how ill I was *

Participants with no previous experience of mental health problems described this as an aggravating circumstance, since they were not able to recognize early symptoms and felt that they were not able to act accordingly to prevent more serious problems. This sometimes led to a dramatic development, where, for example, anxiety was mistaken for cardiovascular problems and ended with visits to the emergency centre or the psychiatric emergency centre:

I hadn’t been feeling well for a long time and then I got a summer job at a shop after graduating from college. […] And then I worked for a few weeks, it felt good, and I was comfortable and enjoying it. But then suddenly, when I got home it felt like I had just died inside, and I just sobbed and sobbed. […] And then I felt like, it felt like I was not alive […] I was completely exhausted. So then my mother called the psychiatric emergency, and I went and got to talk to someone. (IP 5, woman, retail sales worker, service)

This type of dramatic development was a more frequent story among male participants. For some other participants the onset was not as dramatic, and they instead described how they could not function as before, and how they started to make errors at work and how they could no longer manage their work tasks:

I guess it was just that "no, now I cannot take it anymore". You get so tired, and you make so many mistakes at work… You get reports showing your errors, and then it becomes… then you know that there is too much stress and so. (IP 17, man, factory worker, blue-collar worker)

### Additional private life burdens

The participants attributed that work and private life together generated too much negative pressure and that this combination caused their sick leave.

#### Activities besides work add to the overall pressure

It was more common that the work spilled over into participants’ private lives than the opposite, and that participants ceased doing activities that had previously given them energy and satisfaction:

I used to dance once a week, but I stopped because I did not have the energy. (IP 1, woman, after-school leisure teacher, education)

Some participants shared stories about how their private life was the main cause of their sick leave. For a few, this had to do with lifestyle factors, including staying up too late at night to party, extensive exercising or studying in addition to work.

#### Responsibility for care of relatives is a burden *

More common was to be burdened by caregiving responsibilities in the private sphere, which was mostly described by women. Only a few of the participants had children, so care responsibilities were reported as due to parents, siblings, or other relatives being ill or having other difficulties. These difficulties included relatives with severe mental illness, drug problems or physical chronic illness:

As a result, almost all of my summer vacation was spent caring for her even though she has home care. (IP 9, woman, occupational therapist, health care)

#### Problematic upbringing and personal tragedy

In relation to private reasons for sick leave, some participants talked about difficulties from their childhood or personal tragedies from their life, like miscarriage. This was never mentioned as the main reason for the sick leave, but as something that lay at the foundation of their general mental state and therefore contributed to the sick leave.

I’ve never received any support from my own family and I haven’t, I haven’t had a really good relationship with my mother and my father has never been in the picture so there’s a lot of things that come into play I have a pretty shitty background so there’s a lot that comes into play on that front as well. (IP 15, man, chef, service)

#### Poor romantic relationships *

For some of the participants, poor romantic relationships or separations contributed to their mental health problems. Some female participants also described destructive relations which reduced the possibility to recover after a workday, and which was eventually seen as a contributing factor for sick leave:

And all the arguments, all the time. Yes, it was completely crazy. It was like… It was really that relationship that made it really crash, but then it has been a lot at work for a long time. (IP 7, woman, occupational therapist, health care)

## Discussion

The aim of this study was twofold: to investigate 1) perceived causes of sick leave due to CMD among young employees, and 2) differences and similarities among young women and men. The design of the present study put equal emphasis on work, home, and lifestyle situations; however, the results showed that work-related causes dominated strongly for both men and women. Furthermore, the analysis revealed that many of the perceived causes operated in intertwined ways, reinforcing each other. In other words, the young adults described a chain of events that often included a variety of the perceived causes that collectively generated the need to take sick leave.

### The intersection of young age and gender

To analyse the findings by an applied gender perspective focusing on the intersection of young age can help us better understand similarities and differences in experiences expressed by the young men and women. Even in Sweden, often regarded as one of the world’s most gender equal societies, there are different expectations on young men and women [53]. In general, girls are more successful in school and later in academic studies. However, the labour market is segregated both horizontally and vertically and men usually benefit from this segregation. This means that men often have higher salaries, and more often have leading positions. Meanwhile women are to a lager extent employed in low status work sectors dealing with humans rather than in high status sectors like technology or finance [29]. Both young men and women in our study expressed beliefs about that women must work harder to be valued as competent as their male colleagues. This is in line with previous work life research, showing how women must work harder and perform better than men to be valued equally by the employer [54]. The young women spoke more about having high ambitions and taking on too much responsibility. This result aligns with previous studies on the gender gap in adolescents’ mental health, which have shown that Swedish girls experience more educational pressure and perceived more stress [55] and psychological distress [56] in comparison to boys. Male participants encountered more hostile attitudes from managers even if some women also described situations where managers had yelled at them. There were also a few stories from female participants that were examples of sexual or gender-based harassments.

Young adults, regardless of sex, worry about their future in many ways and the labour market is characterised by uncertainty [57]. In our study, the young employees were full of life with high expectations, but lacked the previous experience needed to set limits or prioritize. When they started to feel stressed, they would seldom ask for help. They wanted to be “good” employees and they feared losing their jobs or not having their temporary employment extended. They also shared stories about employers that took advantage of their precarious position in the labour market, making them work long shifts without possibilities to recover. According to some of the stories they even worked illegally many hours according to Swedish law. We would claim that this fear is an expression of real-life situations, where the risk of unemployment [21] and precarious employment among young adults is a common and increasing phenomenon, and is associated with many negative health implications, including mental health problems [11,58,59].

Negative aspects of private life contributing to the participants’ sick leave was more frequently described by women and involved a heavy burden of taking care of very sick relatives or dealing with strained romantic relationships. However, it was not a strong theme in comparison to work-related causes. In other studies, with older women, private burdens have often been identified as an important factor for sick leave related to mental health problems [60]. Given the young age in our study population, private aspects may not yet be a heavy burden. For one thing, only a few of the participants had children, which is often described as a risk factor in sick leave for women [37].

Furthermore, young employees face societal and workplace norms and expectations that state that to be ‘young’ equals being ‘healthy’ and strong, as expressed by both female and male participants. Even though the prevalence of sick leave due to CMD worldwide is high among young adults [18], it is still shameful to talk about mental health and CMD, especially for the young men in our study. This finding coincides with previous research showing that stigma due to masculinity norms prevents young men from seeking help for mental health problems [61]. Masculinity is associated with power, being strong and independent, and the intersecting social categories of age (to be young) and gender (to be male) are reinforcing the risk of not seeking help in times of need [62,63]. One serious implication is that men tend not to seek healthcare for mental health problems to the same extent as women [64]. This could have severe consequences; previous studies have demonstrated that young men with specific mental disorders, (major depression, borderline personality disorder, and substance abuse) are linked to suicide [61].

In a fast spinning world where young people try to find their way, their entrance to the labour market is crucial. To start working life with experiences of sick leave can affect the coming work life negatively [17] and the societal costs of sick leave are significant [5]. Work legislation restricting for example work hours is essential, but according to the study, participants are not always followed by their employers. In the current study, many of the sick leave cases seemed to be possible to avoid if employers had followed the legislation and worked proactively for a better work environment, according to the young adults of the current study. A supportive work environment can, for young employees, include better introduction to tasks and to the workplace, more continuous feedback including help to set limits and prioritize, and an openness from managers to allow the sharing of troublesome experiences that can affect work performance as well as employees’ wellbeing.

### Strengths and limitations

The recommendations from SRQR guidelines and the COREQ checklist were taken into account when designing and conducting this study [65,66] to improve the quality of the study. The proceedings of the study, in terms of research design, data collection and analysis, has essentially followed the protocol without any major changes. As with all qualitative studies, we cannot claim generalisability [44]; we have, however, described the circumstances regarding our study in order to enhance its transferability to other contexts. One central aspect of this study is an applied gender perspective, and the results are strengthened by an equal number of young men and women (12 and 13, respectively) working in sectors dominated by one of the sexes or which is known to have a gender balance.

## Conclusions

An overall finding was that there seems to be more pressure on young women compared to their male counterparts. They express a need to perform better to be regarded as competent as men and they take on more social responsibility at work and in their private lives. These two gendered processes at work and in private life could help understand why young women are on sick leave due to CMD more often than young men. On the other hand, our study confirmed that masculinity norms contribute to difficulties in men asking for help and talking about mental health problems, which can lead to severe problems.

## Supporting information

S1 Checklist COREQ (COnsolidated criteria for REporting Qualitative research) checklist. (PDF)

## References

[1] Axén I, Björk Brämberg E, Vaez M, Lundin A, Bergström G. Interventions for common mental disorders in the occupational health service: A systematic review with a narrative synthesis. International archives of occupational and environmental health. 2020;93(7):823–38. doi: 10.1007/s00420-020-01535-4 32246230 PMC7452923

[2] Lager ACJ, Bremberg SG. Association between labour market trends and trends in young people’s mental health in ten European countries 1983–2005. BMC public health. 2009;9(1):325. doi: 10.1186/1471-2458-9-325 19737380 PMC2748078

[3] de Vries H, Fishta A, Weikert B, Rodriguez Sanchez A, Wegewitz U. Determinants of Sickness Absence and Return to Work Among Employees with Common Mental Disorders: A Scoping Review. Journal of occupational rehabilitation. 2017;28(3):393–417. 10.1007/s10926-017-9730-1 PMC6096498 28980107

[4] Patton GC, Coffey C, Romaniuk H, Mackinnon A, Carlin JB, Degenhardt L, et al. The prognosis of common mental disorders in adolescents: a 14-year prospective cohort study. The Lancet. 2014;383(9926):1404–11. doi: 10.1016/S0140-6736(13)62116-9 24439298

[5] Stansfeld S, Clark C, Bebbington P, King M, Jenkins R, Hinchliffe S. Common mental disorders. NHS digital; 2016.

[6] Steel Z, Marnane C, Iranpour C, Chey T, Jackson JW, Patel V, et al. The global prevalence of common mental disorders: a systematic review and meta-analysis 1980–2013. International Journal of Epidemiology. 2014;43(2):476–93. doi: 10.1093/ije/dyu038 24648481 PMC3997379

[7] Lorentzen T, Bäckman O, Ilmakunnas I, Kauppinen T. Pathways to Adulthood: Sequences in the School-to-Work Transition in Finland, Norway and Sweden. Social Indicators Research. 2019;141(3):1285–305.

[8] Bültmann U, Arends I, Veldman K, McLeod CB, van Zon SKR, Amick Iii BC. Investigating young adults’ mental health and early working life trajectories from a life course perspective: the role of transitions. Journal of epidemiology and community health. 2020;74(2):179–81. doi: 10.1136/jech-2019-213245 31694872 PMC6993030

[9] Keus van de Poll M, Nybergh L, Lornudd C, Hagberg J, Bodin L, Kwak L, et al. Preventing sickness absence among employees with common mental disorders or stress-related symptoms at work: a cluster randomised controlled trial of a problem-solving-based intervention conducted by the Occupational Health Services. Occupational and environmental medicine. 2020;77(7):454–61. doi: 10.1136/oemed-2019-106353 32291291 PMC7306872

[10] Harvey SB, Modini M, Joyce S, Milligan-Saville JS, Tan L, Mykletun A, et al. Can work make you mentally ill? A systematic meta-review of work-related risk factors for common mental health problems. Occupational and Environmental Medicine. 2017;74(4):301–10. doi: 10.1136/oemed-2016-104015 28108676

[11] Toivanen S, Tarantino AO, Emmelin M, Ostergren P-O. Diverting blame to stay sane—young people’s strategies for dealing with the mental health effects of precarious employment: a grounded theory study. BMC public health. 2020;20(1):571. doi: 10.1186/s12889-020-08626-4 32345287 PMC7189722

[12] Vancea M, Utzet M. How unemployment and precarious employment affect the health of young people: A scoping study on social determinants. Scandinavian journal of public health. 2017;45(1):73–84. doi: 10.1177/1403494816679555 27885160

[13] Bjorkenstam E, Helgesson M, Gustafsson K, Virtanen M, Hanson LLM, Mittendorfer-Rutz E. Sickness absence due to common mental disorders in young employees in Sweden: are there differences in occupational class and employment sector? Social psychiatry and psychiatric epidemiology. 2021;57(2). doi: 10.1007/s00127-021-02152-3 34386867 PMC9042979

[14] Helgesson M, Tinghög P, Wang M, Rahman S, Saboonchi F, Mittendorfer-Rutz E. Trajectories of work disability and unemployment among young adults with common mental disorders. BMC Public Health. 2018;18(1):1228. doi: 10.1186/s12889-018-6141-y 30400785 PMC6219052

[15] Harkko J, Virtanen M, Kouvonen A. Unemployment and work disability due to common mental disorders among young adults: selection or causation? European Journal of Public Health. 2018;28(5):791–7. doi: 10.1093/eurpub/cky024 29514230

[16] De Girolamo G, Dagani J, Purcell R, Cocchi A, McGorry P. Age of onset of mental disorders and use of mental health services: needs, opportunities and obstacles. Epidemiology and psychiatric sciences. 2012;21(1):47–57. doi: 10.1017/s2045796011000746 22670412

[17] Eaton WW, Muntaner C, Sapag JC. Socioeconomic Stratification and Mental Disorder. In: Scheid TL, Brown TN, editors. A Handbook for the Study of Mental Health: Social Contexts, Theories, and Systems. 2 ed. Cambridge: Cambridge University Press; 2009. p. 226–55.

[18] OECD. Sick on the Job? 2012.

[19] Kaltenbrunner Bernitz B, Grees N, Jakobsson Randers M, Gerner U, Bergendorff S. Young adults on disability benefits in 7 countries. Scandinavian Journal of Public Health. 2013;41(12_suppl):3–26. doi: 10.1177/1403494813496931 24077622

[20] Försäkringskassan. Sjukfrånvaro i psykiatriska diagnoser, En registerstudie av Sveriges arbetande befolkning i åldern 20–69 år. Sick leave in psychiatric diagnoses, A register study of Sweden’s working population aged 20–69. Includes an English summary.; 2020.

[21] OECD. Mental Health and Work: Sweden. Paris: OECD Publishing; 2013.

[22] Ziaei S, Hammarström A. What social determinants outside paid work are related to development of mental health during life? An integrative review of results from the Northern Swedish Cohort. BMC Public Health. 2021;21(1):1–15. 34847924 10.1186/s12889-021-12143-3 PMC8638423

[23] Christensen A-D, Jensen SQ. Doing intersectional analysis: Methodological implications for qualitative research. NORA-Nordic Journal of Feminist and Gender Research. 2012;20(2):109–25.

[24] Landstedt Gådin KG. Seventeen and stressed–Do gender and class matter? Health Sociology Review. 2012;21(1):82–98.

[25] Heise L, Greene ME, Opper N, Stavropoulou M, Harper C, Nascimento M, et al. Gender inequality and restrictive gender norms: Framing the challenges to health. The Lancet. 2019;393(10189):2440–54. doi: 10.1016/S0140-6736(19)30652-X 31155275

[26] Gender Connell R., health and theory: Conceptualizing the issue, in local and world perspective. Social Science & Medicine. 2012;74(11):1675–83. 21764489 10.1016/j.socscimed.2011.06.006

[27] Keisu B-I, Brodin H, Tafvelin S. On Equal Terms? Gendering Labour Markets, the Organisation of Work, and the Well-Being of Employees. Gendered Norms at Work: New Perspectives on Work Environment and Health, Aligning Perspectives on Health, Safety and Well-Being. Cham: Springer International Publishing; 2021. p. 1–11.

[28] Ellingsæter AL. Scandinavian welfare states and gender (de) segregation: Recent trends and processes. Economic and Industrial Democracy. 2013;34(3):501–18.

[29] Bridges T, Taylor CJ, Robinson S. 10 Connections between Masculinity, Work, and Career Reproduce Gender Inequality, in Men, masculinities and the modern career (Ed. K. Aakvik). 2020:193–216.

[30] Nybergh L, Bergstrom G, Jensen I, Hellman T. Experiences of interventions and rehabilitation activities in connection with return-to-work from a gender perspective. A focus group study among employees on sick leave for common mental disorders. PloS one. 2021;16(6):e0253049–e. doi: 10.1371/journal.pone.0253049 34170934 PMC8232439

[31] Charles M. Deciphering Sex Segregation:Vertical and Horizontal Inequalities in Ten National Labor Markets. Acta Sociologica. 2003;46(4):267–87.

[32] Sverke M, Falkenberg H, Kecklund G, Magnusson Hanson L, Lindfors P. Women and men and their working conditions: the importance of organizational and psychosocial factors for work-related and health-related outcomes. Arbetsmiljöverket; 2017.

[33] Nybergh L, Bergstroem G, Hellman T. Do work- and home-related demands and resources differ between women and men during return-to-work? A focus group study among employees with common mental disorders. BMC public health. 2020;20(1):1914–17. doi: 10.1186/s12889-020-10045-4 33334324 PMC7745371

[34] Lidwall U, Bill S, Palmer E, Olsson Bohlin C. Mental disorder sick leave in Sweden: A population study. Work (Reading, Mass). 2018;59(2):259–72. doi: 10.3233/WOR-172672 29355123

[35] Hausmann R, Tyson LDA, Zahidi S, editors. The global gender gap report 20122012: World Economic Forum Geneva.

[36] Grönlund A, Öun I. Minding the Care Gap: Daycare Usage and the Negotiation of Work, Family and Gender Among Swedish Parents. Social Indicators Research. 2020;151(1):259–80.

[37] Holmlund L, Tinnerholm Ljungberg H, Bültmann U, Holmgren K, Björk Brämberg E. Exploring reasons for sick leave due to common mental disorders from the perspective of employees and managers—what has gender got to do with it? International journal of qualitative studies on health and well-being. 2022;17(1):2054081–. doi: 10.1080/17482631.2022.2054081 35341475 PMC8959517

[38] Holmgren K, Ivanoff SD. Women on sickness absence-views of possibilities and obstacles for returning to work. A focus group study. Disability and Rehabilitation. 2004;26(4):213–22. 15164955 10.1080/09638280310001644898

[39] Boot CR, Bosma AR. How qualitative studies can strengthen occupational health research. Scandinavian journal of work, environment & health. 2021;47(2):91. doi: 10.5271/sjweh.3943 33306131 PMC8114569

[40] Olsson C., Tinnerholm Ljungberg H., Björk Brämberg E., Jensen I., & Nybergh L. (2021). A Gender Perspective on Sick Leave Among Young Adults–Barriers and Resources for Return to Work as Experienced by Young Employees and Managers: A Protocol for a Qualitative Study. International Journal of Qualitative Methods, 20, 16094069211032071.

[41] Kvale S. Doing interviews: Thousand Oaks, Calif. Sage; 2008.

[42] Archibald MM, Ambagtsheer RC, Casey MG, Lawless M. Using Zoom videoconferencing for qualitative data collection: Perceptions and experiences of researchers and participants. International Journal of Qualitative Methods. 2019;18:1609406919874596.

[43] Moser A, Korstjens I. Series: Practical guidance to qualitative research. Part 3: Sampling, data collection and analysis. The European Journal of General Practice. 2017;24(1):9–18. doi: 10.1080/13814788.2017.1375091 29199486 PMC5774281

[44] Patton MQ. Qualitative research & evaluation methods: integrating theory and practice. 4. ed. ed. Thousand Oaks, California: SAGE Publications, Inc.; 2015.

[45] Glise K, Hadzibajramovic E, Jonsdottir I, Ahlborg G. Self-reported exhaustion: A possible indicator of reduced work ability and increased risk of sickness absence among human service workers. International Archives of Occupational and Environmental Health. 2010;83(5):511–20. doi: 10.1007/s00420-009-0490-x 19943058

[46] Lisspers J, Nygren A, Söderman E. Hospital Anxiety and Depression Scale (HAD): some psychometric data for a Swedish sample. Acta psychiatrica scandinavica. 1997;96(4):281–6. doi: 10.1111/j.1600-0447.1997.tb10164.x 9350957

[47] Malterud K. Qualitative research: Standards, challenges, and guidelines. The Lancet. 2001;358(9280):483–8. doi: 10.1016/S0140-6736(01)05627-6 11513933

[48] Guillemin M, Gillam L. Ethics, Reflexivity, and “Ethically Important Moments” in Research. Qualitative Inquiry. 2004;10(2):261–80.

[49] Bourdieu P, Editor. The weight of the world: Social suffering in contemporary society. Oxford: Polity; 1999.

[50] Hsieh H-F, Shannon SE. Three Approaches to Qualitative Content Analysis. Qualitative Health Research. 2005;15(9):1277–88. doi: 10.1177/1049732305276687 16204405

[51] Alfasoft. Nvivio 2022 [Available from: https://www.alfasoft.com/se/.

[52] Elo S, Kääriäinen M, Kanste O, Pölkki T, Utriainen K, Kyngäs H. Qualitative Content Analysis:A Focus on Trustworthiness. SAGE Open. 2014;4(1):2158244014522633.

[53] Gustafsson Sendén M, Klysing A, Lindqvist A, Renström EA. The (Not So) Changing Man: Dynamic Gender Stereotypes in Sweden. Frontiers in Psychology: 2019;10:37. doi: 10.3389/fpsyg.2019.00037 30761034 PMC6363713

[54] Quadlin N. The mark of a woman’s record: Gender and academic performance in hiring. American sociological review. 2018;83(2):331–60.

[55] Wiklund M, Malmgren-Olsson E-B, Ohman A, Bergström E, Fjellman-Wiklund A. Subjective health complaints in older adolescents are related to perceived stress, anxiety and gender—a cross-sectional school study in Northern Sweden. BMC public health. 2012;12(1):1–13. doi: 10.1186/1471-2458-12-993 23158724 PMC3533931

[56] Campbell OLK, Bann D, Patalay P. The gender gap in adolescent mental health: A cross-national investigation of 566,829 adolescents across 73 countries. SSM—Population Health. 2021;13:100742. doi: 10.1016/j.ssmph.2021.100742 33748389 PMC7960541

[57] Tevington P. Privileged to Worry: Social Class, Cultural Knowledge, and Strategies toward the Future among Young Adults. The Sociological Quarterly. 2018;59(2):204–33.

[58] Gunn V, Kreshpaj B, Matilla-Santander N, Vignola EF, Wegman DH, Hogstedt C, et al. Initiatives Addressing Precarious Employment and Its Effects on Workers’ Health and Well-Being: A Systematic Review. International Journal of Environmental Research and Public Health. 2022;19(4):2232. doi: 10.3390/ijerph19042232 35206419 PMC8872425

[59] Canivet C, Bodin T, Emmelin M, Toivanen S, Moghaddassi M, Östergren P-O. Precarious employment is a risk factor for poor mental health in young individuals in Sweden: a cohort study with multiple follow-ups. BMC public health. 2016;16(1):687–. doi: 10.1186/s12889-016-3358-5 27485322 PMC4969669

[60] Holmgren K, Dahlin-Ivanoff S, Björkelund C, Hensing G. The prevalence of work-related stress, and its association with self-perceived health and sick-leave, in a population of employed Swedish women. BMC Public Health. 2009;9(1):73. 19254367 10.1186/1471-2458-9-73 PMC2653036

[61] Lesage AD, Boyer R, Grunberg F, Vanier C, Morissette R, Ménard-Buteau C, et al. Suicide and mental disorders: a case-control study of young men. The American journal of psychiatry. 1994: 151: 1063–1068. doi: 10.1176/ajp.151.7.1063 . 7503818

[62] Levy JK, Darmstadt GL, Ashby C, Quandt M, Halsey E, Nagar A, et al. Characteristics of successful programmes targeting gender inequality and restrictive gender norms for the health and wellbeing of children, adolescents, and young adults: a systematic review. The Lancet Global Health. 2020;8(2):e225–e36. doi: 10.1016/S2214-109X(19)30495-4 31879212 PMC7025324

[63] Rice S, Oliffe J, Seidler Z, Borschmann R, Pirkis J, Reavley N, et al. Gender norms and the mental health of boys and young men. The Lancet Public health. 2021;6(8):e541–e2. doi: 10.1016/S2468-2667(21)00138-9 34332667

[64] Olsson S, Hensing G, Burström B, Löve J. Unmet Need for Mental Healthcare in a Population Sample in Sweden: A Cross-Sectional Study of Inequalities Based on Gender, Education, and Country of Birth. Community Mental Health Journal. 2021;57(3):470–81. doi: 10.1007/s10597-020-00668-7 32617737 PMC7904545

[65] Tong A, Sainsbury P, Craig J. Consolidated criteria for reporting qualitative research (COREQ): a 32-item checklist for interviews and focus groups. International Journal for Quality in Health Care. 2007;19(6):349–57. doi: 10.1093/intqhc/mzm042 17872937

[66] O’Brien BC, Harris IB, Beckman TJ, Reed DA, Cook DA. Standards for Reporting Qualitative Research: A Synthesis of Recommendations. Academic Medicine. 2014;89(9):1245–51. doi: 10.1097/ACM.0000000000000388 24979285

